# LAMA2 Neuropathies: Human Findings and Pathomechanisms From Mouse Models

**DOI:** 10.3389/fnmol.2020.00060

**Published:** 2020-04-23

**Authors:** Stefano Carlo Previtali, Alberto Andrea Zambon

**Affiliations:** ^1^Neuromuscular Repair Unit, Institute of Experimental Neurology (InSpe), Division of Neuroscience, IRCCS Ospedale San Raffaele, Milan, Italy; ^2^Department of Neurology, IRCCS Ospedale San Raffaele, Milan, Italy

**Keywords:** *LAMA2 gene*, neuropathy, human, mousemodel, laminin

## Abstract

Merosin deficient Congenital Muscular Dystrophy (MDC1A), or LAMA2-related muscular dystrophy (LAMA2-RD), is a recessive disorder resulting from mutations in the *LAMA2* gene, encoding for the alpha-2 chain of laminin-211. The disease is predominantly characterized by progressive muscular dystrophy affecting patient motor function and reducing life expectancy. However, LAMA2-RD also comprises a developmentally-associated dysmyelinating neuropathy that contributes to the disease progression, in addition to brain abnormalities; the latter often underappreciated. In this brief review, we present data supporting the impact of peripheral neuropathy in the LAMA2-RD phenotype, including both mouse models and human studies. We discuss the molecular mechanisms underlying nerve abnormalities and involved in the laminin-211 pathway, which affects axon sorting, ensheathing and myelination. We conclude with some final considerations of consequences on nerve regeneration and potential therapeutic strategies.

## Introduction

*LAMA2* encodes the α2 chains of the laminin-211 (also known as merosin), a major component of the basal lamina of Schwann cells and skeletal muscles (Ehrig et al., 1990). Indeed, loss of function mutations of the *LAMA*2 gene in humans, and *the Lama*2 gene in rodents, results in muscular dystrophy, dysmyelinating neuropathy, and brain abnormalities. This results in Merosin-deficient Congenital Muscular Dystrophy (MDC1A, OMIM #607855) also known as LAMA2-related muscular dystrophy (LAMA2-RD).

## LAMA2 Neuropathy in Human Being

Shortly after the identification of the *LAMA2* gene in 1995 (Helbling-Leclerc et al., 1995), abnormalities in the nerve conduction studies of children affected by LAMA2-RD were reported (Shorer et al., 1995). Over two decades later, the clinical significance and pathophysiology of such alterations are yet to be clarified.

The first neurophysiological studies conducted on genetically confirmed patients outlined the high prevalence of mild-to-moderate motor demyelinating neuropathy: deep peroneal nerve motor conduction velocity ranged from 27 to 42 m/s in patients older than 2 years (normal values >42 m/s; 43–57 m/s; Shorer et al., 1995). Although initial reports suggested that compound motor action potential (CMAP) amplitudes and sensory fibers were both preserved in LAMA2-RD, subsequent studies disproved these findings.

Neurophysiological evidence of demyelinating sensorimotor neuropathy can be present as early as 1–6 months of age. With growth, conduction velocities may progressively decrease (Mercuri et al., 1996; Quijano-Roy et al., 2004) along with a reduction of CMAP amplitudes, consistent with a combined axonal and demyelinating polyneuropathy (Brett et al., 1998; Fujii et al., 2011; Verma et al., 2018). Conversely, conduction blocks have not been reported in other studies (Di Muzio et al., 2003; Quijano-Roy et al., 2004; Verma et al., 2018). Although the presence of residual merosin in muscle usually correlates with a milder clinical phenotype and lesser muscle involvement, there is no proven relation with peripheral nerve damage. This may be due to either discordant expression of the laminin-α2 chain in the basement membrane surrounding myofibers and Schwann cells, or the role of compensatory tissue-specific laminin isoforms (see paragraphs below on animal models; Vainzof et al., 1995; Mora et al., 1996; Prelle et al., 1997; Di Muzio et al., 2003).

Muscle and skin biopsies of patients affected by LAMA2-RD display absence of laminin α2 in intramuscular motor nerves (a finding not observed in patients with secondary merosin deficiency), and in skin neural structures, respectively (Tomé et al., 1994; Hayashi et al., 1995; Osari et al., 1996; Marbini et al., 1997; Sewry et al., 1997; Chan et al., 2014).

Morphological data from sensory sural nerve biopsies have been scarcely described in the literature. The few studies available show a reduced number of fibers, especially those of larger caliber (>6–7 μm), and variable myelin diameter. In particular, both focally thickened myelin (tomacula like), and thinner and uncompacted myelin have been reported; the former predominantly in small fibers and possibly at paranodes. Associated findings were shorter internodes and wider nodes of Ranvier (>5 μm), suggesting a disorder in myelinogenesis that resembles murine models (Shorer et al., 1995; Mercuri et al., 1996; Deodatoa et al., 2002; Di Muzio et al., 2003; Quijano-Roy et al., 2004; North et al., 2014). Whilst demyelination and onion bulbs were not observed in sural nerve biopsies, post-mortem pathology of the cauda equina showed clear evidence of ongoing segmental demyelination and remyelination in one case (Hissong et al., 2016). Moreover, one case report described a marked reduction in the number of myelinated axons together with naked axons and increased collagen deposition on electron microscopy (Brett et al., 1998).

It is still not clear to which extent peripheral neuropathy contributes to muscle weakness in patients affected by LAMA2-RD. Absent deep tendon reflexes, distal muscle atrophy and weakness, neurophysiology, and neuropathology studies are consistent with a predominantly dysmyelinating sensory-motor polyneuropathy with some axonal involvement (Mora et al., 1996; Deodatoa et al., 2002; Di Muzio et al., 2003; Verma et al., 2018). However, studies are reporting the preservation of CMAP amplitudes and the absence of neurogenic changes on electromyography (EMG), suggesting that axonal degeneration may be negligible in some patients (Quijano-Roy et al., 2004). It is of course possible that neurogenic defects are somehow masked in these patients by the predominant muscular dystrophy phenotype, or that nerve involvement is prevalent in some mutation types.

Neurophysiology revealed reduced sensory action potentials (SAP) in a few cases (Di Muzio et al., 2003; Quijano-Roy et al., 2004). However, tactile sensation, proprioception, and vibration are usually normal (Chan et al., 2014) or mildly altered (Mora et al., 1996) at clinical assessment.

Overall, the main neuropathophysiological feature of LAMA2-RD seems to be an abnormal maturation of myelin sheets accompanied by segmental demyelination. In murine models, the secondary axonal loss has been extensively described; yet, this is less commonly reported in humans (Brett et al., 1998). Neuropathy could be still a potentially detrimental contributor to disease burden, particularly in patients with partial deficiency where the overall clinical picture is not overshadowed by severe muscle involvement. Finally, although we lack consistent data suggesting progressive axonal loss and clinically significant neuropathy in patients with LAMA2-RD, future therapies might reveal new phenotypes.

## *LAMA2* Neuropathy in Mouse Models

The prototype of LAMA2-RD was first reported in mice of the Bar Harbor 129 Re strain in 1955 (*dystrophic* mice *Lama2*^dy/dy^); this was however limited to the recessive inherited muscular dystrophy phenotype (Michelson et al., 1955). In the 1970s, nerve conduction studies showed functional abnormalities of the peripheral nerve (Papapetropoulos and Bradley, 1972; Bradley, 2008), which was confirmed by subsequent morphological studies. In 1971, Harris observed a reduced number of intramuscular myelinated fibers in *Lama2*^dy/dy^ mice (Harris and Wilson, 1971). This data was confirmed and quantified in the tibialis nerve in 1972 (Harris et al., 1972), and further extended to other nerves and roots by Bradley and Jenkison (1973). Similar findings were described a year later in *Lama2*^dy2J/dy2J^ (Biscoe et al., 1974). The genetic characterization of *dystrophic* mice was achieved two decades after this, when mutations of the Laminin chain α2 gene (*Lama2*) were reported (Xu et al., 1994; Sunada et al., 1995). Recently, mouse engineering by homologous recombination generated further mutants that have almost complete (*Lama2*^dyW/dyW^; Kuang et al., 1998) or a complete lack (*Lama2*^dy3K/dy3K^; Miyagoe et al., 1997) of laminin-211 expression. Finally, a further mutant was generated by ENU-induced point mutation C79R, called *Lama2*^dynfm417/dynfm417^ (Patton et al., 2008). This mouse mutant shows typical *Lama2* muscle and nerve pathology in the presence of normal expression (but not function) of laminin-211. This is likely due to defective high-level organization (3D interactions between different laminin-211 heterotrimers; see Yurchenco and McKee, 2019) and missing interaction with other specific molecular partners.

**Figure 1:**
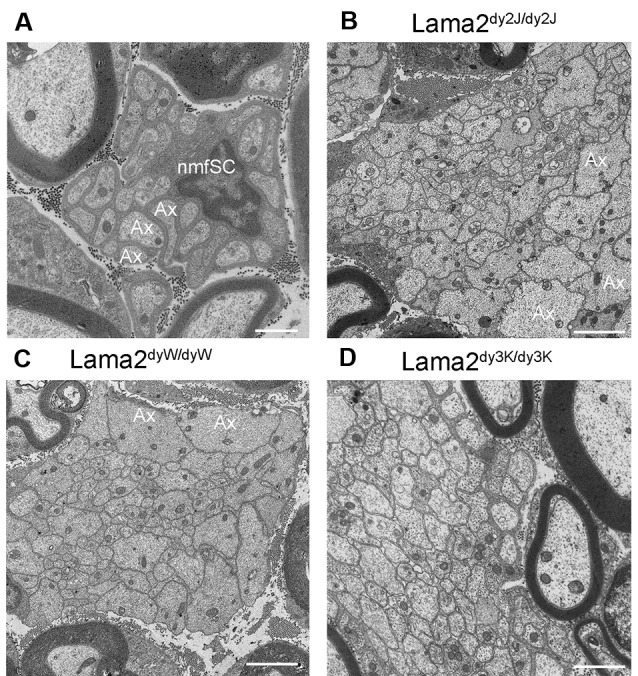
Bundle of unsorted axons in *Lama2* mutants. **(A)** Normal Remak fiber, formed by a non-myelin forming Schwann cell (nmfSC) with unmyelinated axons (Ax); axons are smaller than 1 μm and are well separated and ensheathed by a single non-myelin forming Schwann cell. **(B–D)** Bundle of unsorted, non-ensheathed, and tightly packed axons of different caliber (some are larger than 1 μm, as Ax in figure **B** and **C**), are similarly present in Lama2^dy2J/dy2J^ **(B)**, Lama2^dyW/dyW^ **(C)** and Lama2^dy3K/dy3K^ **(D)** mice. Bar = 1 μm in **(A)**, 2 μm in **(B,C)**, 1 μm in **(D)**.

**Figure 2:**
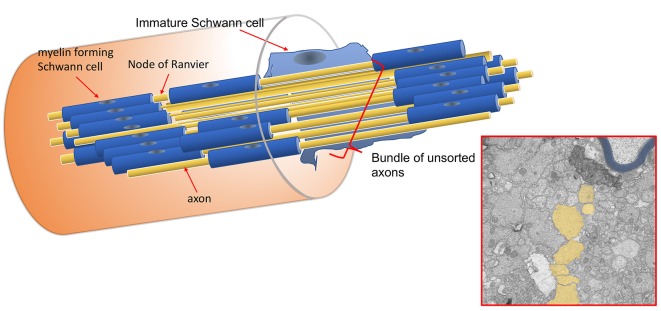
Schematic representation of unsorted axons in *Lama2* nerves. Each axon along its length can be myelinated at one internode and belong to a bundle of unsorted axons in the subsequent one. The electron microscope (E.M.) constitutes a hypothetical cross-section of the nerve fibers in the scheme; unsorted axons are pseudo-colored in yellow, myelinated fibers in blue.

**Figure 3:**
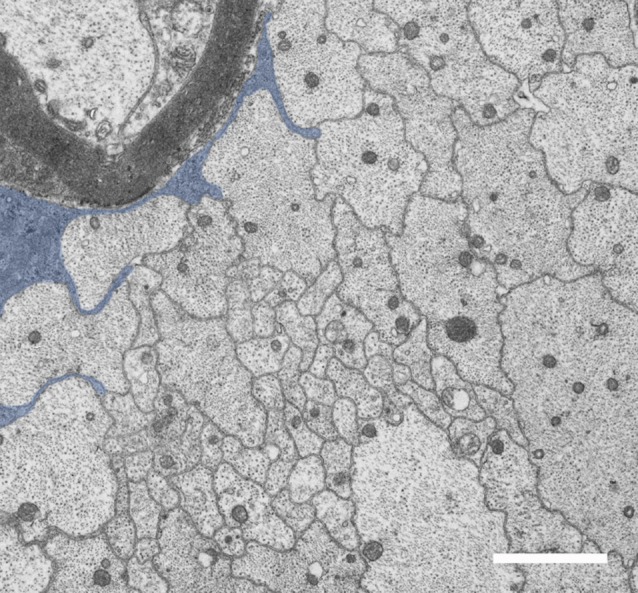
*Lama2* Schwann cells fail to sort axons. Unsorted axons are surrounded by immature Schwann cells that fail to sort them. In this E.M. photograph from Lama2^dy2J/dy2J^ sciatic nerve, the axon protrusions of one immature Schwann cell are pseudo-colored in blue. Bar = 1 μm.

All of these *Lama2* mutants are characterized by progressive muscle wasting and consequent motor impairment, ranging from a less severe phenotype and almost normal lifespan of *Lama2*^dy2J/dy2J^ (and likely *Lama2*^dynfm417/dynfm417^) to the most severe form *Lama2*^dy3K/dy3K^, which die within 3–4 weeks after birth. Peripheral nerves display typical morphological features that are considered prototypic abnormalities of *Lama2* neuropathy. The morphological hallmark is the presence of defective axonal sorting during nerve development resulting in bundles of “naked” unsorted axons (Figures 1, 2 and Supplementary Table S1). This finding is spanning from spinal roots (more consistently) to peripheral nerves, including cranial nerves (Biscoe et al., 1974). These bundles contain several axons of mixed caliber, as well as those larger than 1 μm that should be sorted out and myelinated. Axons are tightly packed and often completely unsheathed or only partially surrounded by Schwann cell protrusions (Figure 3). Axonal sorting is a process that in rodents is completed within a few weeks, starting a couple of weeks before birth and ending around postnatal day 10 (P10). A similar process likely occurs in all vertebrates. In this process, immature Schwann cells surrounding bundles of mixed caliber axons start a physiological process of axon “docking” and “locking” to sort them out of the bundle, as well described and illustrated by Henry Webster in the 1970s and recently reviewed (Feltri et al., 2016). Only axons with a diameter larger than 1 μm are sorted, although the molecular mechanism that sustains their selection (docking) and engagement (locking) is still mostly unclear. In parallel, immature Schwann cells proliferate to match the axon number and to organize a continuous basal lamina around them (Webster et al., 1973; Jessen and Mirsky, 2005). In *Lama2* models, many axon bundles are devoid of Schwann cell processes, although in other Schwann cell processes are visible between axons (Stirling, 1975; Yu et al., 2001; Yang et al., 2005). This also reflects the mouse age as they tend to reduce in size and number with age (at least in long-living *Lama2^dy2J/dy2J.^mice*), suggesting that radial sorting may last longer in mutant mice (Yang et al., 2005 and S. C. Previtali personal observation).

Other nerve abnormalities include a reduced number of myelinated fibers as already reported in the original description of *Lama2*^dy/dy^ mice, including ventral and dorsal roots, tibial and sciatic nerves (Harris et al., 1972; Bradley and Jenkison, 1973; Salafsky and Stirling, 1973). Axon diameters are significantly reduced in *Lama2*^dy2J/dy2J^ (Gawlik et al., 2006) along with a reduced number of myelinated fibers in *Lama2*^dy3K/dy3K^ mice (Yu et al., 2001). Occasional degenerating axons are described in *Lama2*^dy/dy^ mice (Bradley and Jenkison, 1973).

Myelin thickness is reported normal or reduced in many myelinated fibers of *Lama2*^dy/dy^ and *Lama2*^dy2J/dy2J^ mice (Gawlik et al., 2006), in which few fibers (usually of small diameter) may have thicker myelin sheath (Ghidinelli et al., 2017). In *Lama2*^dy3K/dy3K^ nerves, fibers are significantly thinner (Yu et al., 2001).

Nodes of Ranvier are reported wider in *dystrophic Lama2*^dy/dy^ and *Lama2*^dy2J/dy2J^ (Madrid et al., 1975; Jaros and Bradley, 1979; Occhi et al., 2005), whereas they are narrowed in *Lama2*^dy3K/dy3K^ (Yu et al., 2001; Gawlik et al., 2006). Internodes are diffusely shorter in almost all the *Lama2* subtypes (Court et al., 2009 and E. Porrello and S.C. Previtali, unpublished results).

Finally, sensory nerves are sometimes reported as morphologically less affected than motor nerves (Jaros and Jenkison, 1983), although our experience revealed similar findings in motor and sensory roots and nerves (S.C. Previtali, unpublished results).

With regards to other laminin isoforms described in peripheral nerves of *Lama2* mice, the α1 chain (generating laminin-111) is not expressed in wild type nerves, whereas it is reported to be upregulated in sciatic nerves of *Lama2*^dy2J/dy2J^ mice (Previtali et al., 2003b), but absent in *Lama2*^dy3K/dy3K^ (Gawlik et al., 2006). The α4 chains, generating the main laminin isoform in embryonic nerve development laminin-411, is reported to be upregulated in the nerves of Lama2^*dy/dy*^, *Lama2*^dy2J/dy2J^ and *Lama2*^dy3K/dy3K^ mice (Patton et al., 1997; Ringelmann et al., 1999; Yu et al., 2001; Gawlik et al., 2006; Domi et al., 2015). The α5 chain (generating laminin-511) is also modestly upregulated in nerves of *Lama2*^dy/dy^, *Lama2*^dy2J/dy2J^ and *Lama2*^dy3K/dy3K^ mice (Patton et al., 1997; Ringelmann et al., 1999; Gawlik et al., 2006; Domi et al., 2015). Moreover, β1 and γ1 chains, necessary to generate all the laminin isoforms, are normally expressed in all the *Lama2* mutants (Patton et al., 1997; Gawlik et al., 2006).

Accordingly, minor defects in radial sorting are described when Laminin-411 is deleted (Yang et al., 2005), whereas a severe defect is observed when both α2 and α4 chains are ablated (Yang et al., 2005). Similarly, targeted deletion of γ1 chain, impeding the formation of all the above-mentioned laminin isoforms (laminin-111, -211, -411 and -511) results in the most severe and widespread neuropathy characterized by complete lack of myelination and sorting defect (Chen and Strickland, 2003).

## Molecular Mechanism of *LAMA2* Neuropathy

Several laminin-211 receptors have been described so far in peripheral nerves, which activate downstream signaling pathways necessary for proper nerve development, function, and maintenance. Among them, the expression of integrins α6β1, α7β1, α6β4 and dystroglycan has been reported as timely and spatially regulated (Previtali et al., 2003b; Berti et al., 2006), and their effective function confirmed or denied by specifically targeted gene disruption. The G-protein-coupled receptor 126 (GPR126) has been more recently included in this list (Petersen et al., 2015).

Expression studies (Previtali et al., 2003b) showed that β1 integrins are the first ones expressed in nerve development, since early embryonic stages, likely associated with the α6 subunits. Dystroglycan appears later on, around the time of birth. Finally, α7β1 and α6β4 appear in post-natal development. Accordingly, the deletion of the integrin β1 chain resulted in severe neuropathy with axonal sorting defects and dysmyelination (Feltri et al., 2002), thus confirming the role of β1 integrins in this pathway and the pathogenesis of the neuropathy. The Schwann cell deletion of dystroglycan, instead, only mildly impacted nerve development (Saito et al., 2003; Berti et al., 2011), whereas the deletion of both β1 integrins and dystroglycan almost completely impaired axonal sorting and myelination (Berti et al., 2011). Not surprisingly, loss of β4 integrin did not affect nerve development, while it may confer stability to mature myelin in peripheral nerves (Nodari et al., 2008).

More intriguingly, neither loss of α6 nor α7 integrin chains in Schwann cells affected nerve development and function (Previtali et al., 2003a; Pellegatta et al., 2013). While the loss of α7 could be compensated by the presence of other (redundant) β1 integrins (likely α6β1) in post-natal development, it was unexpected that deletion of α6, expressed since early embryonic nerve development, did not affect nerve formation. It was shown that α7 is upregulated, thereby compensating for the absence of α6 during nerve development and that both α6 and α7 integrins should be deleted to impair the ability of Schwann cells to spread and bind laminin-211 or -411 (Pellegatta et al., 2013). However, double α6/α7 integrin mutants showed only a mild phenotype suggesting that other Schwann cell-β1 integrins might also contribute to radial sorting during peripheral nerve development (Pellegatta et al., 2013). Integrin β1 can couple with other α chains described in the peripheral nerve, such as α1, α2, α3, α4, α5, α8, α9, α10 and αv (Lefcort et al., 1992; Milner et al., 1997; Stewart et al., 1997; Previtali et al., 2003b). These β1-integrins bind other extracellular matrix components (i.e., collagen, fibronectin, vitronectin, tenascin, and partially laminins) and may participate in radial sorting of axons or partially compensate for the loss of α6 and α7 in mutant mice. Accordingly, loss of collagen XV aggravates radial sorting defects in laminin-411 null mice (Rasi et al., 2010).

Downstream signaling pathways originating from integrins and/or dystroglycan laminin-211 receptors have been widely, although not exhaustively, investigated. They involve molecules mainly regulating cytoskeleton rearrangement for proper cell polarization and formation of Schwann cell protrusions necessary for axon recognition, sorting, and ensheathment. Among others, they include integrin-linked kinase (ILK), focal adhesion kinase (FAK), Rho (Rac1, Cdc42) and Ras (RalA/B) -GTPases, Profilin, Merlin/neurofibromin (Nf2) and neuronal Wiskott-Aldrich syndrome protein (N-WASP; Benninger et al., 2007; Nodari et al., 2007; Pereira et al., 2009; Jin et al., 2011; Novak et al., 2011; Guo et al., 2013; Grove and Brophy, 2014; Montani et al., 2014; Ommer et al., 2019). Other laminin-211 downstream molecules are instead involved in regulating cell cycle and survival for proper matching of axon-Schwann cell units, such as Cdc42, FAK, Wingless-Integrated (Wnt)/α-catenin, and Jab1 (Benninger et al., 2007; Grove et al., 2007; Grigoryan et al., 2013; Porrello et al., 2014). Most of these molecules and associated pathways have been previously reviewed (Monk et al., 2015; Feltri et al., 2016).

More recently, a strict interaction between laminin-211 and neuregulin 1 type III (NRG1 III), the main signal for peripheral nerve myelination (Nave and Salzer, 2006), has been discovered (Ghidinelli et al., 2017). During nerve development, laminin-211 limits NRG1 III function through the inhibition of protein kinase A (PKA). Loss of laminin-211 would result in overactivation of the NRG1 III pathway resulting in defective radial sorting, inappropriate/premature myelination causing polyaxonal myelination or thicker myelin sheath (Ghidinelli et al., 2017). This would explain the occurrence of hypermyelinated small-caliber fibers in *LAMA2* patients (Shorer et al., 1995; Di Muzio et al., 2003) and mice (Ghidinelli et al., 2017). Whether this effect is mediated by different laminin-211 receptors, and possibly α6α4, remains elusive (Heller et al., 2014).

Finally, studies in zebrafish and mouse mutants showed that GPR126 is required in Schwann cells for myelin expression (Monk et al., 2009) and radial sorting of axons (Monk et al., 2011; Mogha et al., 2013). GPR126 acts as a collagen IV and laminin-211 receptor (Paavola et al., 2014; Petersen et al., 2015), whose interaction promotes receptor cleavage into N-terminal fragment (NTF) and seven-transmembrane containing C-terminal fragment (CTF; Langenhan et al., 2013). The NTF fragment is necessary to guide radial sorting of axons and is generated by the interaction of GPR126 with a sort of (more) “immature” laminin-211 (i.e., low polymerization state), thus keeping the GPR126 receptor “inactive” for myelination (Petersen et al., 2015). Laminin-211 maturation (i.e., polymerization and interaction with other ECM components) switches to GPR126 in “active” state and through CTF can promote cAMP elevation, PKA activation, and thus myelination (Petersen et al., 2015).

## Regeneration in *LAMA2* Neuropathies

Intact Schwann cell basal lamina and correct formation of regenerating tracks of transdifferentiated Schwann cells (known as Bungner bands) is a prerequisite to preserve Schwann cell-axon interaction in successful nerve regeneration after damage (Jessen and Mirsky, 2019). Thus, matrix components of the basal lamina, such as laminin-211, would constitute key elements for nerve regeneration. Accordingly, the expression of laminin-211 and -411 (either as protein or mRNA) are upregulated after nerve damage (Wallquist et al., 2002). Moreover, laminin-211 is well known to promote neurite growth and nerve regeneration (Anton et al., 1994), even as a substrate of artificial nerve graft (Seo et al., 2013).

It is therefore not surprising that *Lama2*^dy/dy^ Schwann cells provide a poor environment for neurite growth *in vitro* (Uziyel et al., 2000). Accordingly, nerve or spinal root damage in *Lama2*^dy/dy^ mice resulted in defective axon regeneration and remyelination (Bray et al., 1983; Uziyel et al., 2000). Defective reinnervation was also observed in *Lama2*^dy2J/dy2J^ mice (Parry and Melenchuk, 1981; S.C. Previtali and E. Porrello, unpublished results), and have been described in mice with conditional inactivation of the laminin γ1 chain, disrupting both laminin-211 and -411 (Chen and Strickland, 2003). Finally, the deletion of the laminin α4 chain did not affect nerve regeneration, suggesting that only laminin-211 (not -411) is necessary for the nerve to regenerate (Wallquist et al., 2005).

In conclusion, although there is no direct evidence in human LAMA2-RD patients, data from animal models suggest that defective nerve regeneration may contribute to the progression of *LAMA2* neuropathy.

## Laminin α2 Chain and Neuromuscular Junctions

At the basal lamina of neuromuscular junctions (NMJs), the laminin α2 chain assembles in trimers with β2 and γ1 forming laminin-221 (Sanes et al., 1990; Patton et al., 1997). Therein, other laminin isoforms are also present, including laminin-421 and -521 (Patton et al., 1997). These three laminin isoforms are essential in establishing and maintaining the structure of NMJs and the alignment of the presynaptic zone (Rogers and Nishimune, 2017). Thus, potentially, loss of laminin chain α2 might affect NMJ formation and function, contributing to the motor phenotype of *LAMA2/Lama2*.

There were no reports in the literature of NMJ abnormalities in *LAMA2* patients, neither in terms of symptoms nor as neurophysiological findings typical of the myasthenic syndrome. Single fiber EMG was reported in one case and described as unremarkable (Chan et al., 2014). *Lama2* mice have been investigated at NMJs. *Lama2* mutants (at least *Lama2*^dy/dy^ and *Lama2*^dy2J/dy2J^) develop smaller post-synaptic junctional folds, partial axon detachment and minor Schwann cell infiltration of the synaptic cleft (Gilbert et al., 1973; Banker et al., 1979; Law et al., 1983; Desaki et al., 1995). However, they normally assemble the presynaptic active zone and properly appose to the acetylcholine receptors (Gilbert et al., 1973). More severe effects on NMJs at pre and post-synaptic zone are instead a consequence of the deletion of laminin chain α4 and/or α5, and particularly in mice devoid of β2 chain (reviewed in Rogers and Nishimune, 2017).

Laminin chain α2 has been also described in the assembly and clustering of acetylcholine receptors, through the interaction with agrin, perlecan, and MuSK (Smirnov et al., 2005). However, acetylcholine receptors seem to be preserved in *Lama2*^dy/dy^ and *Lama2*^dy2J/dy2J^ mice (Banker et al., 1979) and are most likely regulated by laminin α4 and α5 chains (Nishimune et al., 2008).

Loss of laminin α2 in Lama2^dy/dy^ does not affect α4 and α5 expression at the NMJ (Ringelmann et al., 1999). α4 and α5 was instead upregulated in Lama2^dy3K/dy3K^ mice, which otherwise showed normal expression of other NMJ components such as neuronal cell adhesion molecule and utrophin (Miyagoe et al., 1997). Finally, NMJs of *Lama2*^dyNmf417/dyNmf417^ mice showed normal expression of laminin chain α2 (as well as other components) suggesting normal assembly and possibly function (Patton et al., 2008).

All these data suggest that loss of laminin-221 at the NMJ is mostly compensated by laminin-421 and -521, although it cannot be excluded that minor abnormalities described in *Lama2* NMJs might contribute to the motor phenotype and axonal neuropathy in these mutants.

## Potential Treatments for *LAMA2/LAMA2* Neuropathy

Lack of the α2 chains of the laminin-211 in peripheral nerves is responsible for peripheral neuropathy in *LAMA2* disorder. The obvious mechanism to repair this genetic defect would involve gene replacing and/or gene editing, still not feasible therapeutically so far. Gene replacement is mainly limited by the size of the *LAMA2* gene (around 9 Kb), too large to be inserted in useful viral vectors. Gene editing, instead, has been successfully used to repair *Lama2* mutations with the contemporary rescue of the peripheral neuropathy (Kemaladewi et al., 2017). Here, the main limitation is related to the off-target effects of the technique (Tsai et al., 2015).

Other strategies have been used to counteract or prevent the neuropathy. One major finding was the observation that the expression of α1 chain in peripheral nerves improved the neuropathy in *Lama2*^dy2J/dy2J^ mice, including axonal sorting and myelination (Gawlik et al., 2006). This was recently confirmed by employing the CRISPR/Cas9 technology targeting the *Lama1* gene promoter delivered by adeno-associated virus (AAV9; Kemaladewi et al., 2019). Laminin-111 protein was upregulated in muscle and nerves of *Lama2*^dy2J/dy2J^ mice, and specifically, in peripheral nerve, it rescued myelination and nerve conduction velocities (Kemaladewi et al., 2019).

A further strategy for treating *LAMA2/Lama2* disorder is the use of linker proteins mini-agrin and αLNNd. The first one was able to reconnect orphan laminin-211 receptors to the other laminin isoforms expressed in muscle and nerves and the second one to allow laminin polymerization (Yurchenco et al., 2018). These proteins are sufficiently small to be packed into AAV vectors. Accordingly, mini-agrin delivery with AAV9 was able to reach the peripheral nerve promoting the amelioration of axonal sorting and myelination in *Lama2*^dyW/dyW^ treated mice (Qiao et al., 2018). Cell delivery of mini-agrin by mesoangioblasts showed instead efficacy in skeletal muscle but not in peripheral nerves, as these cells could not enter the endoneurium and were stopped in the perineurium of treated mice (Domi et al., 2015). Finally, αLNNd was proven to be effective in promoting myelination in the presence of non-polymerizing laminin isoforms (McKee et al., 2012).

Apoptosis was shown to play a role in the pathology of Lama2 mice (Girgenrath et al., 2004; Dominov et al., 2005), while doxycycline, as well as other tetracycline derivatives, had been reported to inhibit apoptosis in mammalian cells (Davies et al., 2005). Thus, doxycycline was investigated in *Lama2*^dyW/dyW^ mutants where it showed amelioration of muscle and nerve pathology (Girgenrath et al., 2009; Homma et al., 2011). Although the doxycycline mechanism of action in nerves of *Lama2* mutants remains vague, it might be linked to reduced cell death of immature Schwann cells and amelioration of Schwann cell differentiation (Homma et al., 2011). Moreover, is possible that doxycycline acts through different mechanisms in different tissues.

Glatiramer acetate (GA), an agent for immune modulation, has been shown to significantly improve mobility and muscle strength in the *Lama2*^dy2J/dy2J^ mice (Dadush et al., 2010). Nerve conduction velocities were also reported significantly increased in these treated mice, suggesting a valuable effect of the drug on *Lama2* neuropathy (Rabie et al., 2019).

## Author Contributions

SP and AZ wrote the manuscript and prepared figures.

## Conflict of Interest

The authors declare that the research was conducted in the absence of any commercial or financial relationships that could be construed as a potential conflict of interest.
